# Eye Injuries in Warfare: The Reports at the Ophthalmological Congress

**Published:** 1915-05-01

**Authors:** 


### EYE INJURIES IN WARFARE.

### The Reports at the Ophthalmological Congress.

The annual Congress of the Ophthalmological Society of the United Kingdom was held on Thursday, Friday, and Saturday, April 22 to 24, under the presidency of Mr. F. Richardson Cross, of Bristol, and was well attended, members being present from all parts of the kingdom. A full clinical afternoon took place at the Royal London Ophthalmic Hospital.

During the proceedings at the Royal Society of Medicine the Nettleship Medal and Prize (in memory of Mr. Edward Nettleship, the eminent ophthalmic surgeon, recently deceased) were presented to Mr. E. Treacher Collins, in recognition of his work for the advancement of ophthalmology.

### Ophthalmic Injuries in Warfare.

Mr. Walter H. H. Jessop, in introducing this subject, dealt chiefly with the cases he had seen from the present war on land, at St. Bartholomew's and No. 1 Base Hospitals. He said that the steel or cupro-nickel mantle of the bullet was often chipped, and a fragment might perforate the cornea, or embed itself in its substance. The initial high velocity of the bullet might be lost by ricocheting from a stone, and he had found that wounds so produced were more serious than direct ones. As most of the fighting was taking place in trenches, there was a greater proportion than usual of head and face injuries, as they were the parts most exposed to the enemy. There had been a few instances of spent bullets entering the orbit, and remaining there without bursting the eyeball; they were probably ricochet. Such cases often showed intraocular hæmorrhages, choroido-retinal changes, and detachment of retina, generally complete. The most distressing ocular injuries, usually caused by enfilading fire, were those in which the bullet passed transversely through both orbits. There must already have been at least fifty such cases. If it was found that a wound had been made in the orbital roof, the brain substance would fill it up; and as sepsis could not be prevented it was better to leave well alone. When the orbit and eyeball were much damaged, it was better to foment the wound and keep it as clean as possible with sodium chloride solution, delaying removal of the eyeball until the parts were less inflamed, always having in mind the risk of ophthalmitis should the other eye be a good one. In the case of both eyes being destroyed, it was better to wait until the danger of sepsis had passed, or until the sepsis was much reduced, before removing the destroyed eyeballs, owing to the risk of meningitis.

He thereupon proceeded to describe an operation devised by Colonel W. T. Lister to avoid opening the nerve sheath. Eyelid injuries he found to be very common, and almost always accompanied by injuries of the eyeball. Usually the lids became very œdematous, hence sutures should not be inserted into them. Conjunctivitis did not seem to have been prevalent in this war. In the Franco-German War, 55 per cent. of cases of eyeball injury developed sympathetic ophthalmitis; in the American Civil War the percentage was 16.14. In the present war he had not seen or heard of a single case of the kind, which he attributed to the great care exercised in diagnosis and treatment, and especially in carrying out early enucleation of a ruptured globe.

He said he had seen only one case of bullet wound implicating ocular nerves; and that case he described. It was made by a ricochet bullet. The bullet and shrapnel wounds of the skull vault were generally depressed, and there was a great frequency of papillœdema in such cases; the appearances were those usually noted to follow pressure. Papillœdema was probably present in all cases of vault injuries with increased intracranial pressure; it was not usually seen when that pressure remained normal. Fractures of the skull near the occipital lobes were often accompanied by hemianopsia. At the beginning of the war there were many cases of complete blindness following severe explosions; but sight returned, at periods up to a week. There was always a bilateral involvement in these cases. Some cases were deaf; in rarer instances the patient was dumb. Three of the men in question had extreme blepharospasm, so that for days they were unable to open their eyes. The initial shock in these cases produced loss of consciousness; and when this passed off it left the patient with loss of sight, of hearing, or of speech, but of temporary duration.

### The Outstanding Feature.

Colonel W. T. Lister, whose contribution was communicated by Mr. Jessop, said the outstanding features of the ophthalmic injuries in the present war were their severity, and the impossibility, in most cases, of employing conservative surgery. Still, there was a great deal of work to be done. He grouped the cases into the following classes. Contusions, wounds, cases with foreign body in the eye, functional cases, and head cases. These he considered *seriatim*. In concussion cases, if the bullet did not go through the orbit, the fundus showed no concussion changes. Rupture of the choroid occurred fairly frequently, and the lesion might be star-like or arranged in definite groups. The remainder of the retina showed some pallor, due to *commotio retinæ*. Penetration of the eye with small foreign bodies was a very unsatisfactory group of cases to deal with. Many of the particles did not give a sensible $x$-ray shadow; and even if they did, many of the fragments were non-magnetic. Two factors increased the poor outlook in operation: (1) the frequent presence of more foreign bodies than had been diagnosed; (2) the friability of the foreign bodies, so that they broke off when caught in the forceps. It was difficult to know what to do in the case of a foreign body in the eye when the sight remained useful. He believed that, even if an eye contained a foreign body, if there were no prolapse or entanglement of the uvea or lens capsule, the risk of sympathetic ophthalmitis was *nil*. Though the eye would probably degenerate, useful vision might persist for a considerable time. If signs of irritation did not abate, he would remove the eye. Foreign bodies in the cornea were often very troublesome to remove. When the foreign body was deeply embedded, he found a Beer's knife most useful.

Colonel Lister then went on to discuss, in some detail, functional cases, and quoted some striking instances. In head injuries papillœdema occurred in slightly over 50 per cent. of the cases. but two-thirds of these cases had merely blurring or slight swelling. The greatest swelling occurred in association with cerebellar abscess. He did not attach much diagnostic importance to hæmorrhages or exudations; and the presence of papillœdema did not point to the immediate necessity of operation; it was increasing swelling which was the significant factor. He regarded cases of occipital injury as of very great interest, as from a study of them important light might be cast on the cortical representation of the fundus. The facts arrived at by Dr. Gordon Holmes and himself from the examination of a number of cases were: (1) When the lesion occurred over the occipital cortex of only one side, the result was either hemianopsia or homonymous quadrantic defect on the opposite side; (2) when the bullet passed through both occipital lobes there was at first complete blindness, but in several cases this gradually cleared centripetally after an interval varying from hours to days, leaving a central scotoma. He regretted his observations were not fuller, but after a short time in France or Flanders cases were sent over to England.

### "Explosion Injuries."

Captain A. W. Ormond followed. He recounted three cases of traumatic amblyopia following the explosion of shells. The first was that of a soldier who was in the retreat from Mons, and, after entire rest and "feeding up," recovered entirely. A second case was associated with an error of refraction, which had led to the idea that recovery had not taken place, while the third has not yet regained his former sight, although wounded three months or more ago. It was pointed out how difficult these cases are to diagnose, and how important it is to take the psychology of the men into consideration. He described the frequency of "explosion injuries" peculiar to the war, the result of hand grenades, shrapnel, or high-explosive shells. Numerous cases had occurred in which, as a result of a general explosion, small fragments of metal or dirt had been forced through the tunics of the eye; these had sometimes been overlooked, and later on, the man having complained of pain and inflammation, iritis had been correctly diagnosed, but the possibility of a foreign body being present in the eye had been unrealised, and sympathetic trouble might have occurred. Captain Ormond related two such cases, but, fortunately, the speedy removal of the eye prevented sympathetic trouble supervening.

With regard to the vexed question of removing an eye containing a foreign body he strongly recommended that no general dogmatic statement should be made on this very debatable point, as every case must be considered individually. He deprecated relying solely on the result of the *x*-ray examination, because in several of the cases the foreign body was not discovered until a second or third photograph had been taken (sometimes not even then). The history, the nature of the inflammation, if any, and the amount of vision should be taken into consideration when coming to a decision.

### Cases at St. Dunstan's.

The author submitted short notes of twenty-eight blinded soldiers, giving brief histories of each, with the main points of ophthalmic interest which they presented. Most of these men are now at St. Dunstan's, Regent's Park; thirteen of them have no perception of light at all, and the sight of those who have vision ranges from a mere perception of light to the capacity to count fingers in one part of their field; eighteen were the victims of bullet wounds which in most cases traversed the front part of the head from side to side; four were wounded by shrapnel, one by a hand grenade, and the rest by explosion. The exact nature of these explosions the men themselves were unable to state. One, however, was due to a bullet hitting the magazine of the man's own rifle, which exploded in his face.

Captain Ormond remarked on the extraordinary malignancy of these bullet wounds, several of the men, when looked at, appearing to be absolutely fit, often with both eyes intact, and having a natural appearance. The scar of the entrance and exit wounds was almost impossible to locate. The author also referred to a case of serious corneal ulceration due to the gonococcus, which was probably caused by infection from a towel which had been used by a number of men; partial recovery ensued. Captain Ormond concluded with an account of some cases seen from the camps in and around London.

